# Beyond Body Mass, Beyond Adulthood: The Ontogeny of Sexual Size Monomorphism

**DOI:** 10.1002/ajpa.70257

**Published:** 2026-05-04

**Authors:** Gabrielle L. Bueno, Stacey R. Tecot, Rebecca J. Lewis

**Affiliations:** ^1^ Department of Anthropology The University of Texas at Austin Austin Texas USA; ^2^ School of Anthropology University of Arizona Tucson Arizona USA

**Keywords:** body size, lemurs, reproductive strategies, sexual dimorphism, sexual selection

## Abstract

**Objectives:**

Contest competition for mates and female reproductive energetics influence body size and sexual dimorphism across many primates; nevertheless, some monomorphic species defy these patterns. These deviations may reflect biological anthropology's focus on body mass and adult size as defining features of sexual dimorphism. Using ontogenetic data, we test whether sexual monomorphism in adult body mass necessitates monomorphism across all traits and evaluate whether mate competition and female energetics explain sex‐specific developmental patterns and adult morphology in a monomorphic primate.

**Materials and Methods:**

We built sex‐specific growth curves using generalized additive mixed models to examine levels of sexual dimorphism in 14 morphological measurements across development in Verreaux's sifaka ( *Propithecus verreauxi* ) at Ankoatsifaka Research Station in western Madagascar.

**Results:**

Sifaka exhibited male‐biased dimorphism in upper arm and thigh circumference at adulthood and across growth, but not in other contest‐related traits. Adult females had significantly longer hindlimbs and thighs and exhibited bimaturism and increased growth rate in these and several other traits.

**Discussion:**

We found limited support for our hypotheses that female reproductive energetics or male–male contest competition drive adult size, sex differences, and growth trajectories in Verreaux's sifaka. However, male–male contest competition likely drives male‐biased dimorphism in muscle mass, and longer female hindlimbs may represent an adaptation to infant carrying, reflecting a species‐specific suite of dimorphic traits. This study demonstrates that sexual dimorphism exists at finer scales even in monomorphic species, and that adult size and sex differences are the result of a mosaic of selective pressures acting on individual traits.

## 1 Introduction

A range of factors (e.g., resource availability, climate, diet, foraging patterns, predation risk, and life history strategy) shape a species' adult body size and growth patterns, including age at adult size, growth rate, and growth duration (Andersson 1994; Bergmann 1847; Dunham et al. 2013; Fleagle 1985; German and Hochberg 2020; Gordon 2006a, 2006b; Harcourt and Schreier 2009; Leigh 1995; Sailer et al. 1985; Shea 1986; Shine 1989; Stearns 1992; Temerin et al. 1984). Within a species, males and females may experience these selective pressures differently, which can result in sexual dimorphism in adult size (Badyaev 2002; German and Hochberg 2020; Leigh 1992). Sexual dimorphism at adulthood is caused by variation in growth duration (bimaturism) and/or variation in growth rates during the developmental period (Jarman 1983; Leigh and Shea 1996; Leigh and Terranova 1998; Shea 1985, 1986). Studying adult sex differences and sex differences in developmental trajectories can help reveal selective drivers for these traits.

Adult sexual size dimorphism can arise due to female reproductive requirements. The “fecundity advantage hypothesis” (Darwin 1871; Shine 1988) posits that larger female body size allows for greater reproductive output. In mammals, larger females are thus hypothesized to birth larger, more robust infants (e.g., Charnov and Berrigan 1993; Gordon 2006b; Kuzawa and Bragg 2012; Püschel et al. 2025; Ralls 1976). By contrast, the “reproductive constraints hypothesis” argues that females gain greater benefits from smaller body size, likely due to the associated energetic efficiency (Cassini 2020; Clutton‐Brock et al. 1977; Gordon 2006a; Lindenfors 2002). The lower metabolic costs of small body size promote greater survival and reproduction in smaller females during times of scarcity, resulting in male‐biased body size dimorphism (Gordon 2006a). While the predicted direction of dimorphism differs, these female‐driven hypotheses predict that females and males exhibit different adult body size and growth trajectories. If the body size of one sex is larger at adulthood, that sex must grow for a longer duration and/or at a higher rate than the other sex. Importantly, if constrained or driven by reproduction, the growth of female body size is expected to cease before or upon the onset of reproduction (Charnov and Berrigan 1993; Leigh and Shea 1995).

Sex differences in intrasexual contest competition can also lead to adult sexual size dimorphism. Because larger and stronger individuals are more likely to win physical fights, adult sexual size dimorphism arises when one sex experiences higher levels of intrasexual contest competition than the other (Andersson 1994; Darwin 1871; McDonald et al. 2005; Plavcan 2001). In some primate studies, this expectation for body size has been simplified to just apply to males and male–male contest competition (e.g., Cassini 2020; Clutton‐Brock et al. 1977; Crook 1972; Kappeler and van Schaik 2004), but this approach fails to consider both that females are an equal component in the sexual dimorphism equation (Gordon 2006a, 2006b; Plavcan 2001, 2011), and that female primates also experience intrasexual competition (Isbell 1991; Koenig 2002; Plavcan et al. 1995). Greater levels of intrasexual contest competition between males compared to females are thus expected to lead to male‐biased dimorphism, while greater levels of intrasexual contest competition between females are expected to lead to female‐biased dimorphism (Andersson and Iwasa 1996; Emlen and Wrege 2004; Rosvall 2011). In primates, high levels of male–male contest competition for mates are often hypothesized to explain male‐biased sexual size dimorphism, particularly in anthropoids (Clutton‐Brock 1985; Leutenegger and Kelly 1977; Plavcan 2001, 2012). Additionally, males in these species exhibiting male‐biased sexual dimorphism attain their larger adult size via increased growth duration and/or growth rate relative to females (Leigh 1995; Leigh and Terranova 1998). If adult male size is driven by male–male contest competition for mates, males are expected to reach adult size at the same age that they start competing seriously for mates (e.g., Leigh 1995; Leigh et al. 2005). For example, male mandrills ( *Mandrillus sphinxus* ) enter the dominance hierarchy at age 9 years, the same age at which they reach adult size in body mass, canine length, testicular volume, and adult coloration (Setchell and Dixson 2002; Setchell et al. 2006). Relatively greater levels of male intrasexual contest competition are not expected to directly influence female adult size or growth trajectory, but strong positive selection for male size may indirectly influence female size via correlated response or selection for females big enough to birth the larger males (Plavcan 2011).

Body size is not the only trait that can improve fighting ability, and thus, unequal levels of intrasexual contest competition can also lead to sexual dimorphism in traits that increase the probability of winning intrasexual physical conflicts. Indeed, male‐biased dimorphism in canine length occurs in many primates, particularly catarrhines (Plavcan 2001, 2012; Leutenegger and Kelly 1977). Furthermore, levels of sexual dimorphism in body mass are not always correlated with levels of sexual dimorphism in other traits (Gustafsson and Lindenfors 2004; Kay et al. 1988; Oxnard 1983; Plavcan 2001; c.f. Wood 1976). In mandrills, which exhibit some of the greatest levels of male‐biased sexual dimorphism in body mass across primates, male reproductive success correlates more closely with canine size than with body mass (Leigh et al. 2008). Sexual dimorphism also occurs in other features that may contribute to male fighting ability, such as body length, sagittal crest height, and femoral length in western gorillas ( *Gorilla gorilla* : Breuer et al. 2007; Taylor 1997), and forelimb, chest, and thigh circumferences in thick‐tailed greater galagos ( *Otolemur crassicaudatus* : Leigh et al. 2024). Consequently, male–male contest competition may select for a variety, or even a suite, of traits related to fighting ability in primates, not just body mass, and the traits most responsive to contest competition may vary across species.

The study of sexual dimorphism in biological anthropology has primarily focused on sex differences in body mass at adulthood (e.g., Cassini 2020; Clutton‐Brock and Harvey 1977; Kappeler 1990; Leigh 1992; Leutenegger and Kelly 1977; Plavcan 2011, 2012). Adult body mass is a useful metric in understanding primate sex differences (Jungers 1985; Leigh 1992) and can play a decisive role in male–male contest competition, particularly in species exhibiting extreme levels of sexual dimorphism, such as catarrhines (Cassini 2020; Plavcan 2001, 2012). However, the focus on body mass and on adult size as the defining features of sexual dimorphism hinders the field's understanding of the proximate and ultimate causes of sex differences by treating complex organisms as a single, static measurement. Body mass is essentially an amalgamation of every morphometric measurement (Leigh 1992) and can thus obscure variation at smaller scales. Body mass additionally fluctuates across seasons, environments, and physiological states (King et al. 2011; Leigh 1992; Lewis and Kappeler 2005; Mariotto et al. 2024; Pereira 1993; Richard et al. 2000). Sex differences in other morphological traits, such as limb length (e.g., carnivores: Morris and Carrier 2016; *Giraffa tippelskirchi* : Cavener et al. 2024; *G. gorilla* : Breuer et al. 2007; macropodids: Richards et al. 2015; *P. verreauxi* : Lawler et al. 2005), muscle mass (e.g., *Canis familiaris* : Pasi and Carrier 2003; *G. gorilla* : Barel Hooge et al. 2024; Zihlman and McFarland 2000; *Mus musculus* : Cooper et al. 2020; *O. crassicaudatus* : Leigh et al. 2024), and canine size (e.g., anthropoids: Leutenegger and Kelly 1977; canids: Gittleman and Valkenburgh 1997; *Hippopotamus amphibius* : Shannon et al. 2021; *M. sphinxus* : Leigh et al. 2008; Moschidae: Jarman 1983; platyrrhines: Kay et al. 1988), may also have a greater impact on contest competition than body mass alone. Furthermore, both the adult traits and the development of these traits are under selection (Badyaev 2002; Fedigan 1982; Leigh et al. 2024; Setchell and Lee 2004; Shea 1986; Turcotte et al. 2022). Even some primate species that exhibit sexual monomorphism in body mass as adults follow different growth trajectories depending on sex (Leigh 1992; Tennenhouse 2015), suggesting males and females of these species face different selective pressures on body size that can only be uncovered by examining ontogeny (Leigh 1992). Together, these observations suggest that, while adult body mass provides useful information in the study of primate sexual dimorphism, additional research and greater resolution is needed to better understand the selective mechanisms driving primate sex‐biased size differences.

Verreaux's sifaka ( *Propithecus verreauxi* ) represents an interesting species with which to study primate sex differences in adulthood and across development. Strepsirrhines are key for understanding the evolution of sexual size differences in primates because levels of sexual size dimorphism are often reported to not adhere to the same relationships with body size or social factors that are seen in anthropoids (Kappeler 1990; Plavcan 2001; Smith and Cheverud 2002; Weckerly 1998). For example, male‐biased sexual size dimorphism is strongly correlated with high levels of polygyny and male–male contest competition in anthropoids (Cassini 2020; Leutenegger and Cheverud 1982; Plavcan 2001), but most strepsirrhines exhibit sexual size monomorphism despite high levels of polygyny (Kappeler 1990). Proposed explanations for why lemurs specifically do not conform to this pattern include Madagascar's strong seasonality constraining growth, thus precluding bimaturism and sexual size dimorphism (Leigh and Terranova 1998; Pereira 1993; cf. Tennenhouse 2015), and selection for agility over body size in male–male contest competition (Clutton‐Brock 1985; Kappeler 1990; Lawler et al. 2005). Adult Verreaux's sifaka exhibit sexual size monomorphism or moderate female‐biased sexual size dimorphism across different populations (Lewis and Kappeler 2005; Richard et al. 2000), and levels of sexual dimorphism in different traits vary across *Propithecus* (Glander et al. 1992; King et al. 2011; Ravosa et al. 1993). Verreaux's sifaka exhibit high levels of competition for mates and resources (Bueno and Lewis 2024; Kappeler and Fichtel 2024; Richard 1992). Males compete for mates via lengthy arboreal chases, and if one male catches the other, the pair will fall to the ground and wrestle (Brockman 1994; Lawler et al. 2005). These chases may explain positive selection for hindlimb length and thigh shape and balancing selection for body mass within male sifaka (Lawler et al. 2005). Physical fights can additionally involve cuffing, biting, and hitting (Brockman 1994; Richard 1992). Verreaux's sifaka also have a long developmental period for their body size relative to other primates (Godfrey et al. 2004; Richard et al. 2002), and their 5‐year period of development (Bueno 2022; Richard et al. 2002) allows for a range of possible ontogenetic trajectories.

The aim of our study was to examine potential drivers of levels of sexual dimorphism in Verreaux's sifaka by examining sex differences in multiple traits throughout development. We first tested the fecundity advantage hypothesis (Darwin 1871; Gordon 2006b; Kuzawa and Bragg 2012; Püschel et al. 2025; Ralls 1976). If adult size and growth in sifaka are driven by selection for larger female size due to reproductive benefits, we predicted that female sifaka have a larger overall adult size than males. We further predicted that females exhibit longer growth duration and/or higher growth rates in all traits, and that females reach adult size before or at the average age of first reproduction (6 years: Bronikowski et al. 2016; Voyt et al. 2019). We next tested the reproductive constraints hypothesis (Clutton‐Brock et al. 1977; Gordon 2006a; Lindenfors 2002). If adult size and growth in sifaka are driven by constraints on female size, we predicted that female sifaka have an overall smaller adult size than males. We additionally predicted that females exhibit shorter growth duration and/or lower growth rates in all traits, with growth ceasing before or upon the onset of reproduction. Finally, we tested the male contest hypothesis. If adult size and growth in sifaka are driven by an inequality in intrasexual contest competition, with males experiencing greater levels of intrasexual contest competition than females, we predicted that adult sifaka exhibit male‐biased sexual dimorphism in physical traits related to fighting, including hindlimb length, thigh length, forelimb length, maxillary canine length, thigh circumference, upper arm circumference, and body mass. These traits aid males in competition that involves chasing, grappling, cuffing, and biting (Li et al. in revision). One caveat for these predictions is that previous research in this species suggests body mass is under stabilizing selection in males (Lawler et al. 2005), and hence lack of support for this prediction would not necessarily negate the hypothesis. We further predicted that males exhibit either longer growth duration and/or higher growth rates in these traits, and that males reach adult size in these traits at the same age they start competing for mating opportunities.

Verreaux's sifaka are reported to be fairly monomorphic in body mass (Lewis and Kappeler 2005; Richard et al. 2000), hence we also considered the possibility that sifaka do not exhibit size dimorphism in other physical traits or growth patterns. This lack of sex differences is expected when either (1) females and males experience similar selective pressures (e.g., metabolic or competitive) or (2) the sexes experience different selective pressures but reach similar adult sizes.

## 2 Methods

### 2.1 Site and Study Population

Ankoatsifaka Research Station is located within Kirindy Mitea National Park, a dry, deciduous forest located in western Madagascar at approximately (20°47′17″′ S, 44°10′0″ E). Verreaux's sifaka group sizes in this population range from 2 to 11 individuals, and group compositions are highly variable (Leimberger and Lewis 2017). Sifaka live in societies with a female‐biased power structure (Brockman 1994; Lewis et al. 2022) and mate polygynandrously (Brockman 1999). They can reach sexual maturity at age 3 years but do not regularly mate or successfully produce offspring until at least age 5 years of age (Kappeler and Fichtel 2012; Lawler et al. 2003; Richard et al. 2002; Voyt et al. 2019), leading to disagreement among researchers as to whether Verreaux's sifaka should be categorized as adults starting at age 3 years or at age 5 years. Because the analyses in this paper were in part intended to clarify age at adulthood, we thus did not define age at adulthood a priori.

We measured all animals in June or in early July prior to July 8th, approximately 1–2 months before each individual's birthdate. All sifaka at Ankoatsifaka are born between July and September each year, with 67% of recorded births occurring in July and 92% in July and August (Lewis, unpub. data). Consequently, ages were categorized as the age the individual was approaching just after measurement. For example, measurements from a lemur that was 4 years and 11 months old in July of 2016 were categorized as age 5 years in 2016 in all analyses.

One unavoidable shortcoming of studies utilizing wild animals is age estimation. Researchers cannot know the exact age of subjects born before a study begins or outside of the study area, and thus some subjects' ages must be estimated based on various aspects of their physical condition, such as dental eruption, tooth wear, and body condition (e.g., Breuer et al. 2009; King et al. 2011). The benefit of long‐term field studies, such as the Sifaka Research Project at Ankoatsifaka, is that extensive data exist on individuals along with their birth date, often accurate to within days or weeks. However, most of our morphometric data for individuals with known birthdates only include individuals younger than age 6 years. Because age 6 years is 1 year later than most estimates of when Verreaux's sifaka reach adulthood (Kappeler and Schäffler 2008; Lawler et al. 2003; Lewis and van Schaik 2007; Richard et al. 2000; cf. Kappeler et al. 2009), we included individuals of adult size and body condition but of unknown age in age categories 6 years and above. Age categories 1 year through 5 years only included individuals with known birth years.

Because the sample size of individuals with known birth dates above age 6 years was sparse, we populated it using data from individuals known to be adults, but whose exact birth year was unknown. In this study, individuals who entered the dataset as adults, determined by dental eruption, tooth wear, body condition, weight, and nipple condition for females, were assigned an age estimate of 5 years at first capture. To be conservative, we discarded data from the first capture for each adult of unknown age for the present study, and we only included data from these subjects starting at their second capture, when they were known to be at least 6 years of age (Table S1). Because substantially smaller sample sizes in the older (> 8 years) known age categories of our dataset might skew the growth curves, we only used the added data to determine an asymptote for each measurement after the point at which all individuals had reached adulthood and to calculate sexual differences at adulthood. To ensure a non‐arbitrary age cutoff, we also ran all analyses using only data from individuals with known birth years at age categories 6, 7, and 8 years, but the results did not vary.

### 2.2 Data Collection

As part of the long‐term Sifaka Research Project, Verreaux's sifaka at Ankoatsifaka were anesthetized and examined annually from 2006 to 2019 (for detailed methods see: Rasambainarivo et al. 2014), excluding 2009 due to Cyclone Fanele (Lewis and Bannar‐Martin 2012). Subjects were anesthetized by remote dart containing tiletamine and zolazepam (15–20 mg/kg) at a distance of 3–6 m by a professional darter using a blow pipe or, infrequently, a CO_2_ injection rifle. We collected body mass using a spring balance and linear measurements (Table 1) using a tape measure. The linear measurements collected using a tape measure in this study include tail‐crown length, hindlimb length, thigh length, leg length, forelimb length, arm length, forearm length, and tail length (Table 1). We measured upper arm and thigh circumferences (Table 1) using a tape measure as a proxy for musculature (Lawler et al. 2005). We measured maxillary canine length using a digital caliper. The same researcher (RJL) collected all measurements each year between 2006 and 2019. Due to variable levels of animals' sedation, we were only able to collect a subset of the morphometric measurements for some subjects. However, we recorded body mass at every capture. All subjects were held for several hours in a cloth bag in the shade until they had fully recovered and were released at the site of capture, within 50 m of their group (located using radio telemetry), or at the center of their home range if the entire group was sedated and released simultaneously. Measurements were adapted from Glander et al. (1992; Table 1).

**TABLE 1:** Morphometric variables.

Measurement	Description
Body mass	Measured in kilograms using a spring balance
Tail‐crown length	Base of the tail to the most anterior point on the head with the head in a normal position
Hindlimb length	From groin to the calcaneal tuberosity *Note that this excludes the hindfoot
Thigh length	From the superior surface of the greater trochanter to the middle of the patella, taken with the knee bent
Leg length	From the superior surface of the calcaneal tuberosity to the middle of the patella, taken with the knee bent
Thigh circumference	Circumference of the midpoint of the upper leg
Forelimb length	From the axillary region to the proximal edge of the friction pad nearest the wrist. *Note that this excludes the forefoot
Arm length	From the lateral margin of the acromion process of the scapula to the olecranon process, taken with the elbow bent
Forearm length	From the midcarpal joint to the olecranon process, taken with the elbow bent
Upper arm circumference	Circumference of the midpoint of the upper arm
Chest circumference	Circumference of the thorax with the tape measure positioned just inferior to the right and left axillae
Tail length	Measured on the dorsal side of the tail from the tip of the tail to the junction at the base of the tail with the lower back. The tail is extended straight out behind the animal
Maxillary canine length	Crown height from the gingival margin to the apex of the crown on its lateral side. The values of the upper left and upper right were averaged to produce one measurement. If one canine was broken, then only the measurement from the other unbroken canine was used
Intermembral index	(Forelimb length/hindlimb length)*100

*Note:* Adapted from Glander et al. (1992).

### 2.3 Study Subjects

From 2006 to 2019 we captured 104 different sifaka (*N*_Females_ = 44). Because some individuals were captured and measured multiple times (maximum number of recaptures = 8), we captured individual sifaka a total of 248 times. Data were discarded from 32 individual sifaka (*N*_Females_ = 12) across 37 captures (*N*_Females_ = 13) that could not be assigned an age based on our conservative age‐estimation methods described above. Our analyses thus used data from 89 individual lemurs (*N*_Females_ = 39) across 211 captures (*N*_Females_ = 101), described in Tables S1 and S2.

To run *t*‐tests examining levels of sexual size dimorphism in adults 6 years and older, morphometric data of each of the 14 dimensions (Table 1) from 42 adults (116 captures, *N*_Females_ = 62, *N*_Male_ = 54) were averaged within individuals. This collation resulted in one average adult value of each dimension for each individual, for measurements from a total of 42 adults (*N*_Females_ = 17, *N*_Male_ = 25). We pooled pregnant (*N* = 47) and nonpregnant (*N* = 23) females because body mass was not significantly different between these groups (*t*(68) = 1.08, *p* = 0.28).

### 2.4 Data Analysis

#### 2.4.1 Growth Curves

We built a growth curve of each morphometric dimension using generalized additive mixed models (GAMMs) using the mgcv package (Wood 2011) in R (R Core Team 2020). GAMMs are ideal for examining growth because they can flexibly model nonlinear growth trajectories while accounting for repeat measures and unbalanced sampling across individuals. We used GAMMs to model the developmental trajectory of 14 linear dimensions (Table 1) across ages 1–10 years and over (10+). To model growth over age for each sex, we included age as a smooth term with sex included in the term as a factor “by” variable. Including sex as a by‐variable of age allows the model to create separate smooths, or growth curves, for each sex, which can then be compared with each other within the model. We added sex separately as a fixed effect to control for identifiability constraints (Simpson 2017). Subject ID was included as a random effect to control for individual variation and repeated measures. Smoothing parameters were selected using the REML method (Wood 2011) to minimize risks of overfitting and over‐smoothing. We plotted growth curves using ggplot2 (Wickham 2016).

#### 2.4.2 Growth Duration

To examine growth duration, and thus age at adult size for each sex, we calculated the first derivatives and confidence intervals of the smooths in each GAMM using the gratia package (Simpson 2022) in R (R Core Team 2020). The first derivative of a given point on a GAMM smooth is the slope of the growth curve at that point and indicates the velocity or rate of change at that age. To determine the average age at which each sex reached adult size in a dimension, we calculated the *x*‐value or age at which the first derivative first reached zero, with a 95% confidence interval (Simpson 2014, 2016) as a derivative value of 0 translates to cessation of growth. We additionally visually examined the growth curves plotted by each GAMM to confirm results. We compared the age at growth cessation for both males and females to determine whether there was any bimaturism. Growth duration differences are described in years; however, because measurements were only collected once a year, true variation likely exists at a finer grain and may vary from the reported ages by several months.

#### 2.4.3 Growth Rate

To compare growth rate for each dimension at each age between males and females, we compared the smooth functions for males and females produced by each GAMM following Rose et al. (2012). Using the gratia package in R (Simpson 2022), we produced a graph plotting the difference in trends, meaning the difference in growth rates, between males and females across growth for each dimension, along with 95% pointwise confidence intervals. Any point at which the plotted trend diverges significantly from zero indicates a difference in growth rate between males and females.

#### 2.4.4 Adult Sexual Dimorphism

To determine rates of sexual size dimorphism for each dimension in adults, we ran two‐sample independent *t*‐tests between all adult males and females using base R (adults were defined as age 6 years and older, based on the results from growth duration analyses). We initially classified adults as 5 years and older. However, our initial examinations of growth curves, average dimensions, and *t*‐test results suggested that data from 5‐year‐old sifaka differed substantially enough from data of sifaka aged 6 years and older to warrant splitting these age categories. We thus classified adults as 6 years and older. To ensure sample size and *p* values were not inflated, and because linear dimensions should not vary after adulthood, we only included each individual once, using the average of each dimension taken across each year they were sampled as adults to reduce the effects of measurement error. We additionally considered significance values for the parametric coefficient of sex in each of the GAMMs, which report the significance of the partial effect of sex across the growth curves. We plotted sex‐specific density distributions of each morphological trait using ggplot2 (Wickham 2016).

### 2.5 Ethical Note

All capture protocols and field research were approved by Madagascar National Parks, the Institutional Animal Care and Use Committee at the University of Texas at Austin, and Madagascar's Ad Hoc Committee for Flora and Fauna (CAFF/CORE). All research complied with the legal requirements of Madagascar and adhered to the guidelines set forth by the American Society of Primatologists Principles for the Ethical Treatment of Non‐Human Primates. All permit numbers are included in a supplemental file.

## 3 Results

### 3.1 Sexual Dimorphism in Growth Duration

**FIGURE 1:**
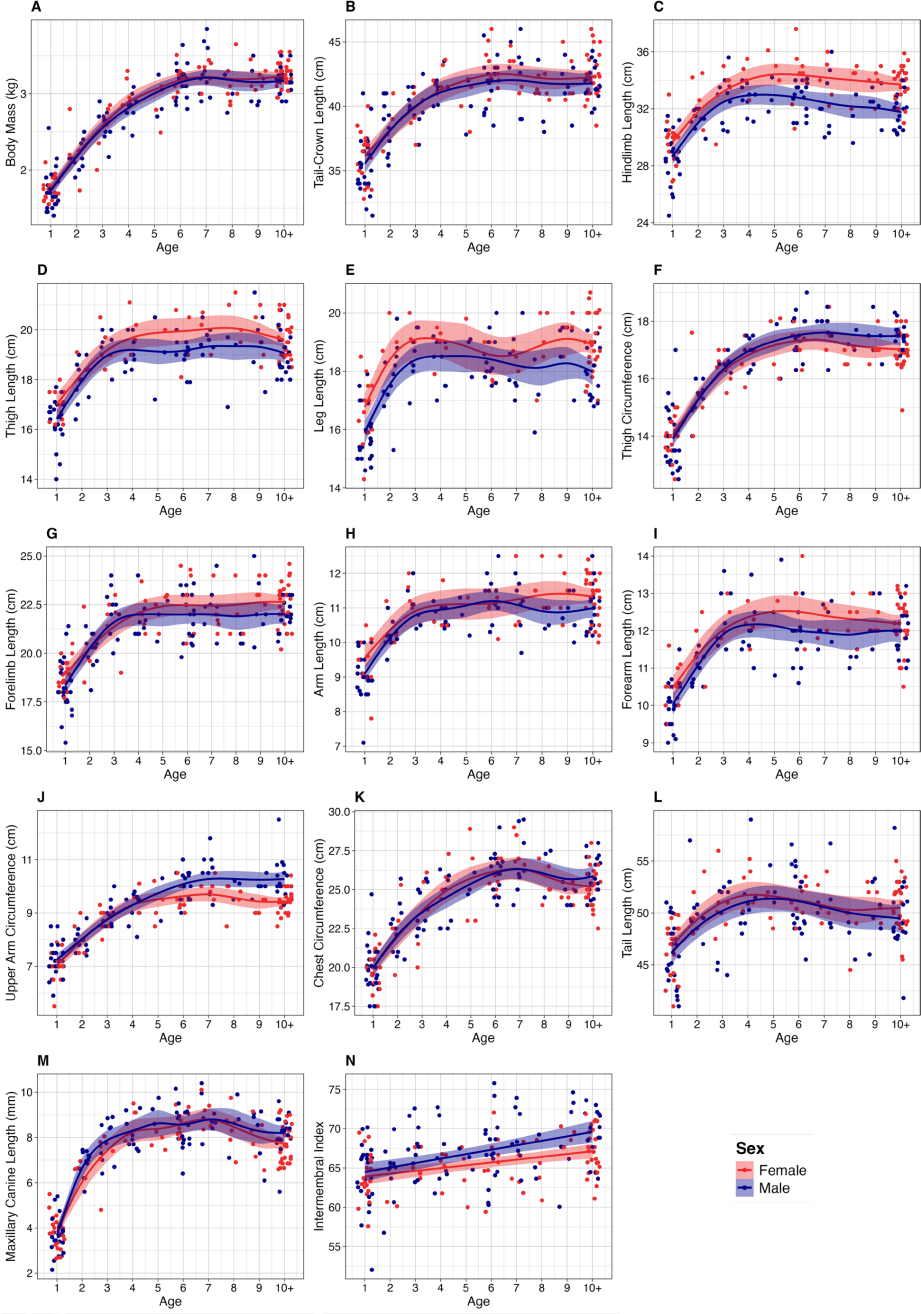
Growth curves over age (in years) separated by sex for the following 14 measurements: (A) body mass, (B) tail‐crown length, (C) hindlimb length, (D) thigh length, (E) leg length, (F) thigh circumference, (G) forelimb length, (H) arm length, (I) forearm length, (J) upper arm circumference, (K) chest circumference, (L) tail length, (M) maxillary canine length, and (N) intermembral index. Each point represents a single individual. Growth curves are plotted as GAMM smooths.

**FIGURE 2:**
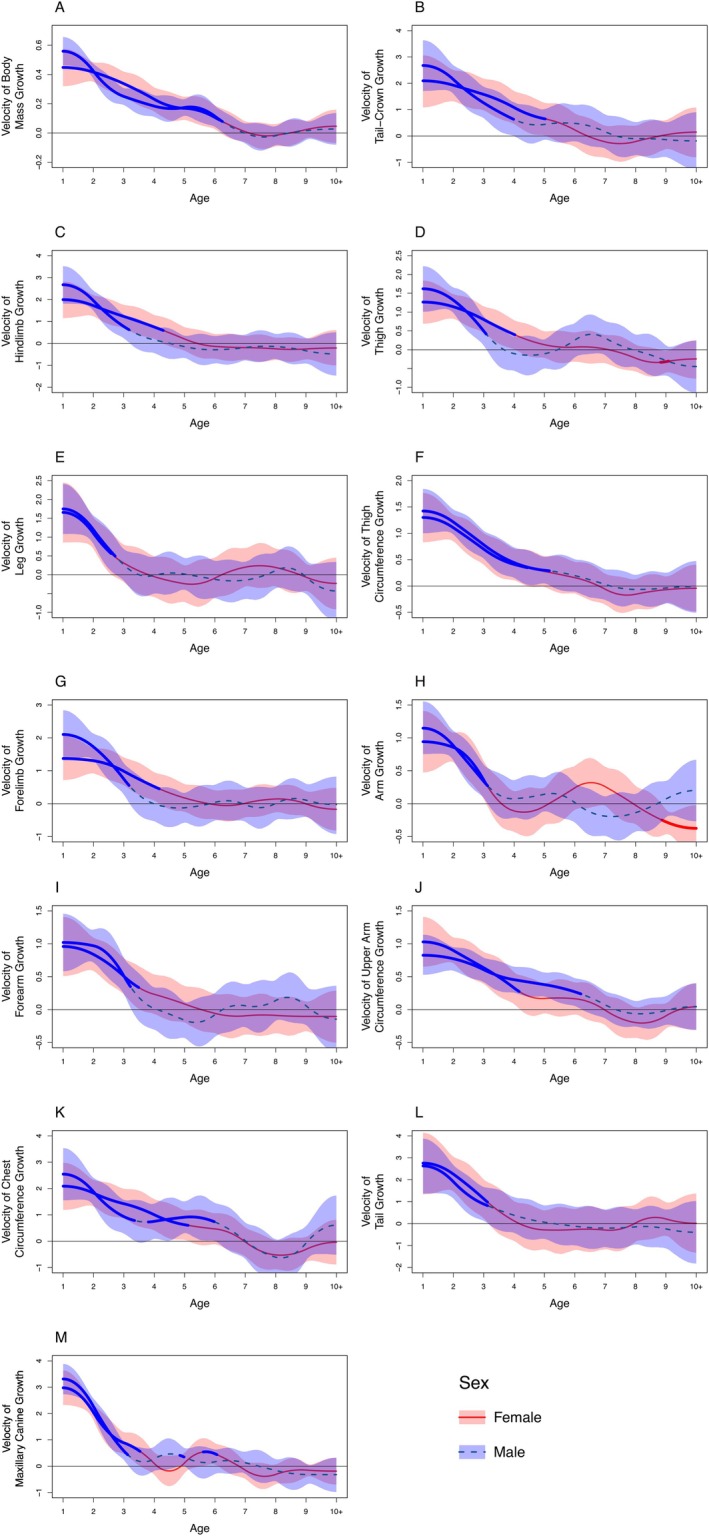
Velocity of growth, depicted by the first derivatives of each GAMM, of the following by age (in years) for males and females: (A) body mass, (B) tail‐crown length, (C) hindlimb length, (D) thigh length, (E) leg length, (F) thigh circumference, (G) forelimb length, (H) arm length, (I) forearm length, (J) upper arm circumference, (K) chest circumference, (L) tail length, and (M) maxillary canine length. The horizontal line at zero indicates a slope of zero on the growth curves (Figure 1). The area above the horizontal line represents positive growth and the area below the horizontal line represents negative growth. When the slope is positive and significantly different from zero, that is, when the growth rate of a given measurement is not zero and the animal is still growing, the derivative line for each sex is highlighted in blue. The point on the *x* axis at which the blue highlight ends, when the slope is not significantly different from zero, is the age at which growth stops. When the slope is negative and significantly different from zero, that is, when the physical trait is shrinking, the derivative line is highlighted in red. The lighter colored shading, blue for males and red for females, represents the 95% confidence interval.

In all dimensions, excluding intermembral index, both male and female sifaka exhibited steady initial increases for the first few years, before tapering off and eventually plateauing at adult dimensional values (Figure 1A–N). Both males and females reached adult body mass at age 6 years (Figure 1A and Table 4). Females exhibited longer duration of growth in tail‐crown length, hindlimb length, thigh length, and forelimb length (Figure 2B–D,G). Females grew for 1 year longer than males in all these dimensions, reaching adult skeletal size at age 5 years while males reached adult skeletal size at age 4 years (Table 4). Males exhibited bimaturism in thigh circumference and upper arm circumference and continued growing 1 and 2 years after female growth cessation in these dimensions, respectively (Figure 2F,J and Table 4). Males and females showed no significant difference in growth duration for body mass (Figure 2A), leg length (Figure 2E), arm length (Figure 2H), forearm length (Figure 2I), chest circumference (Figure 2K), tail length (Figure 2L), or maxillary canine length (Figure 2M and Table 4). Intermembral index increased in both males and females throughout the recorded age span, and the average intermembral index for males was higher than the average intermembral index for females at all ages, but this difference did not reach significance until age 5 years (Figure 1N).

### 3.2 Sexual Dimorphism in Growth Rates

**FIGURE 3:**
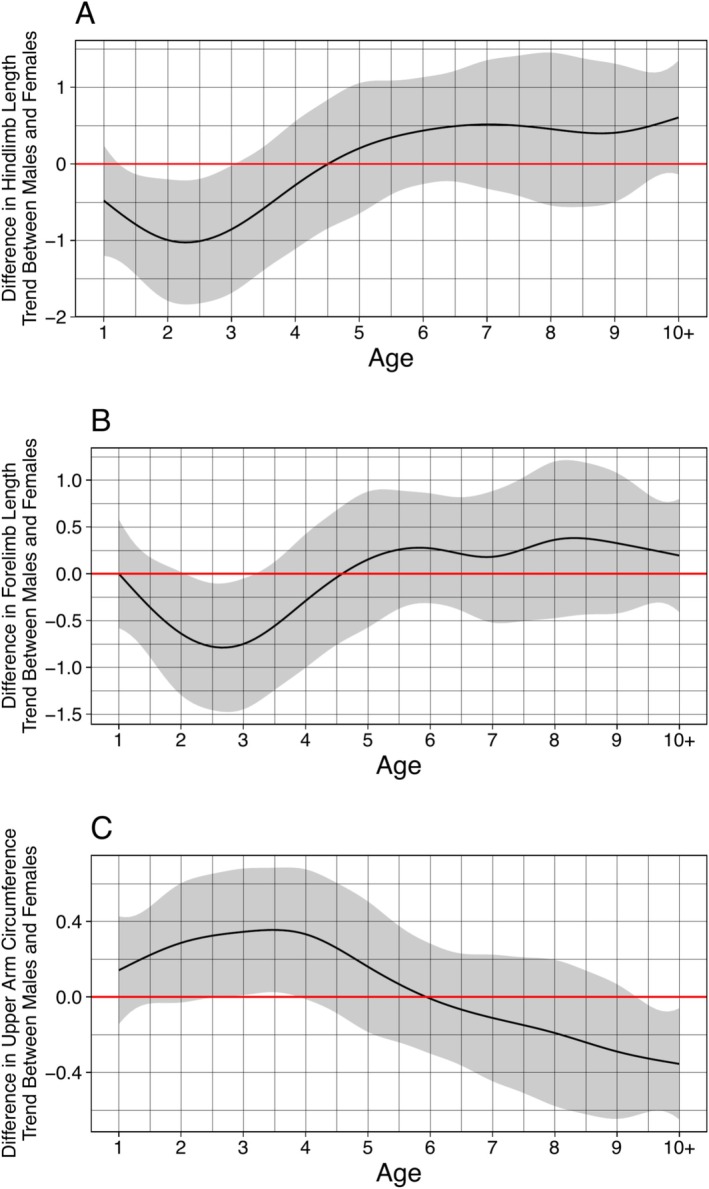
Difference in growth rates between males and females by age for (A) hindlimb length, (B) forelimb length, and (C) upper arm circumference. The red horizontal line at zero indicates no significant difference in growth rate. When the confidence interval excludes zero at any age, the slope of the growth curves and thus rate of growth for a given measurement differs between males and females at that age. The area above the red horizontal line indicates male growth rate is higher than female growth rate, and the area below the red horizontal line indicates female growth rate is higher than male growth rate.

Female sifaka exhibited faster growth rates than males in hindlimb length at ages 2 and 3 years, and in forelimb length at age 3 years (Figure 3A,B). Growth rate in upper arm circumference was greater in males than in females at age 3 years and in the 10+ years age category (Figure 3C). Males and females exhibited no difference in growth rate at any age for body mass, tail‐crown length, thigh length, leg length, thigh circumference, arm length, forearm length, chest circumference, tail length, or maxillary canine length.

### 3.3 Sexual Dimorphism in Adult Sifaka

Adult females had significantly longer hindlimbs (*t*(38) = 3.54, *p* = 0.001) and thighs (*t*(36) = 2.04, *p* = 0.049) than adult males (Table 2). The difference between female and male leg length approached significance, with females exhibiting a nonsignificant trend towards longer legs (*t*(37) = 1.89, *p* = 0.067). Adult males had significantly larger thigh circumference (*t*(39) = −3.04, *p* = 0.004) and upper arm circumference (*t*(37) = −4.85, *p* < 0.001) than adult females (Table 2). Adult males additionally had a significantly higher intermembral index than females (*t*(37) = −2.29, *p* = 0.028). All of these sex differences were also significant in the GAMM output, with the exception of thigh circumference (Table 3).

**TABLE 2:** Means and standard deviations of 14 morphometric measurements of Verreaux's sifaka by age category.

Age (years)	Body mass (kg)				Tail‐crown length (cm)				Hindlimb length (cm)			
	*N*_F_	*N*_M_	Females	Males	*N*_F_	*N*_M_	Females	Males	*N*_F_	*N*_M_	Females	Males
1	20	22	1.74 ± 0.15	1.72 ± 0.28	20	23	36.0 ± 1.9	35.3 ± 2.3	20	22	29.5 ± 1.4	28.5 ± 1.8
2	4	11	2.25 ± 0.44	2.18 ± 0.18	4	11	38.6 ± 1.9	38.6 ± 2.1	4	10	33.0 ± 1.8	31.3 ± 0.7
3	6	10	2.54 ± 0.32	2.59 ± 0.20	6	10	39.6 ± 1.7	40.1 ± 1.6	6	10	33.1 ± 1.9	32.7 ± 1.7
4	5	9	3.04 ± 0.20	2.79 ± 0.20	5	9	42.4 ± 0.9	41.4 ± 1.1	5	9	34.0 ± 1.2	32.8 ± 1.0
5	4	3	2.86 ± 0.28	2.92 ± 0.29	4	3	40.4 ± 1.8	39.9 ± 2.1	4	3	34.8 ± 1.0	32.9 ± 1.2
6+	17	25	3.22 ± 0.18	3.17 ± 0.24	17	25	42.3 ± 1.7	41.9 ± 1.9	**24**	**49**	**33.9 ± 1.4**	**32.3 ± 1.4**

Age (years)	Thigh length (cm)				Leg length (cm)				Thigh circumference (cm)			
	*N*_F_	*N*_M_	Females	Males	*N*_F_	*N*_M_	Females	Males	*N*_F_	*N*_M_	Females	Males
1	12	21	16.9 ± 0.6	16.4 ± 0.9	14	21	16.6 ± 1.1	15.8 ± 1.0	20	23	14.0 ± 0.7	13.9 ± 1.1
2	3	11	18.5 ± 0.6	18.0 ± 0.8	3	11	19.2 ± 0.7	17.8 ± 1.2	4	11	15.5 ± 1.5	15.23 ± 0.6
3	4	7	19.4 ± 0.9	19.3 ± 0.7	4	7	19.0 ± 0.7	18.5 ± 0.9	6	9	16.4 ± 0.6	16.5 ± 0.2
4	5	8	19.7 ± 0.9	19.1 ± 0.7	5	8	18.9 ± 0.6	18.3 ± 0.8	5	9	16.7 ± 0.7	16.9 ± 0.5
5	1	3	20.0 ± NA	18.9 ± 1.7	1	3	19.8 ± NA	18.6 ± 0.7	4	2	17.2 ± 1.1	17.0 ± 1.3
6+	**15**	**23**	**19.7 ± 0.9**	**19.2 ± 0.9**	15	24	18.9 ± 0.9	18.2 ± 1.0	**16**	**25**	**17.1 ± 0.6**	**17.5 ± 0.6**

Age (years)	Forelimb length (cm)				Arm length (cm)				Forearm length (cm)			
	*N*_F_	*N*_M_	Females	Males	*N*_F_	*N*_M_	Females	Males	*N*_F_	*N*_M_	Females	Males
1	20	23	18.9 ± 0.8	18.3 ± 1.4	12	21	9.5 ± 0.7	9.1 ± 0.7	14	21	10.3 ± 0.6	10.0 ± 0.6
2	3	11	20.2 ± 2.0	20.2 ± 0.9	3	11	10.5 ± 0.6	10.2 ± 0.4	3	11	11.7 ± 1.0	11.0 ± 0.4
3	6	10	21.7 ± 1.4	22.1 ± 1.3	4	7	11.5 ± 0.4	11.1 ± 0.6	4	7	12.3 ± 0.5	12.4 ± 0.8
4	5	8	22.2 ± 1.1	21.7 ± 1.0	5	8	10.9 ± 0.6	10.8 ± 0.3	5	8	12.2 ± 0.5	12.0 ± 0.8
5	4	3	22.4 ± 0.9	21.8 ± 1.1	1	3	11.3 ± NA	10.8 ± 0.6	1	3	12.8 ± NA	12.4 ± 1.6
6+	*16*	*25*	*22.6 ± 1.2*	*22.0 ± 1.2*	15	23	11.3 ± 0.7	11.1 ± 0.7	*15*	*24*	*12.3 ± 0.7*	*12.0 ± 0.7*

Age (years)	Upper arm circumference (cm)				Chest circumference (cm)				Tail length (cm)			
	*N*_F_	*N*_M_	Females	Males	*N*_F_	*N*_M_	Females	Males	*N*_F_	*N*_M_	Females	Males
1	19	23	7.0 ± 0.5	7.2 ± 0.6	20	23	19.9 ± 1.2	19.9 ± 1.6	20	23	46.5 ± 2.7	45.7 ± 3.0
2	4	11	8.1 ± 0.8	7.9 ± 0.4	4	11	22.6 ± 2.4	22.5 ± 1.6	4	11	50.9 ± 2.7	50.0 ± 2.6
3	6	10	8.7 ± 0.5	9.0 ± 0.5	6	10	23.3 ± 2.3	23.6 ± 1.4	6	10	50.8 ± 2.9	49.8 ± 3.7
4	5	9	9.6 ± 0.4	9.2 ± 0.6	5	9	25.5 ± 1.1	23.8 ± 1.9	5	9	52.8 ± 2.1	51.1 ± 3.2
5	4	2	9.3 ± 0.7	9.5 ± 1.1	4	3	25.5 ± 3.0	25.3 ± 0.7	4	3	50.4 ± 1.7	52.5 ± 1.9
6+	**16**	**25**	**9.5 ± 0.5**	**10.2 ± 0.6**	16	25	25.6 ± 1.3	26.0 ± 1.4	16	24	50.6 ± 2.2	50.2 ± 3.12

Age (years)	Maxillary canine length (mm)				Intermembral index			
	*N*_F_	*N*_M_	Females	Males	*N*_F_	*N*_M_	Females	Males
1	20	23	3.8 ± 0.8	3.6 ± 0.8	20	22	64.1 ± 3.1	64.0 ± 4.2
2	4	11	6.6 ± 0.3	6.9 ± 0.6	3	10	62.1 ± 3.0	64.4 ± 3.6
3	6	10	7.1 ± 1.2	7.8 ± 0.9	6	10	65.5 ± 1.8	67.6 ± 3.3
4	5	9	8.7 ± 0.7	8.2 ± 0.7	5	8	65.3 ± 2.1	66.6 ± 3.9
5	4	3	7.8 ± 0.4	9.2 ± 0.6	4	3	64.3 ± 3.4	66.2 ± 1.4
6+	17	25	8.1 ± 0.9	8.4 ± 0.9	**16**	**24**	**66.2 ± 2.0**	**68.2 ± 3.5**

*Note:* Adult results are pooled in the category “6+”. Bold font indicates significant differences based on results from *t*‐tests and the partial effects of GAMM smooths. Italic font indicates significant differences based on results from the partial effects of GAMM smooths alone, but not *t*‐tests. Analyses were only run in adults aged 6+. All variables were normally distributed. Reported data for adults in the age 6+ category in this table include measurements from all animal captures; however, as reported in the Section 2, we included each adult in the age 6+ category only once in each *t*‐test, using the average of each dimension measured across adulthood to ensure *p* values were not inflated.

**TABLE 3:** Results for estimates of parametric coefficients and effective degrees of freedom for smooth terms for 14 morphometric measurements and age by sex in Verreaux's sifaka (GAMMs).

Response variable	Body mass			Tail‐crown length			Hindlimb length		
Parametric coefficients	Estimate	SE	*p*	Estimate	SE	*p*	Estimate	SE	*p*
Intercept	2.79	0.04	< 0.001	40.42	0.26	< 0.001	32.74	0.21	< 0.001
Sex (M)	−0.05	0.05	0.29	−0.43	0.35	0.22	−1.35	0.28	< 0.001

Smooth terms	edf		*p*	edf		*p*	edf		p
Age by sex (F)	4.79		< 0.001	3.49		< 0.001	3.77		< 0.001
Age by sex (M)	5.34		< 0.001	4.00		< 0.001	4.70		< 0.001
Adjusted *R* ^2^	0.872			0.654			0.611		

Response variable	Thigh length			Leg length			Thigh circumference		
Parametric coefficients	Estimate	SE	*p*	Estimate	SE	*p*	Estimate	SE	*p*
Intercept	19.04	0.13	< 0.001	18.39	0.15	< 0.001	16.19	0.09	< 0.001
Sex (M)	−0.57	0.18	0.001	−0.78	0.19	< 0.001	0.16	0.13	0.2

Smooth Terms	edf		*p*	edf		*p*	edf		*p*
Age by sex (F)	3.62		< 0.001	4.27		< 0.001	4.01		< 0.001
Age by sex (M)	5.07		< 0.001	4.93		< 0.001	4.29		< 0.001
Adjusted *R* ^2^	0.645			0.527			0.797		

Response variable	Forelimb length			Arm length			Forearm length		
Parametric coefficients	Estimate	SE	*p*	Estimate	SE	*p*	Estimate	SE	*p*
Intercept	21.44	0.16	< 0.001	10.79	0.11	< 0.001	11.79	0.09	< 0.001
Sex (M)	−0.47	0.21	0.027	−0.27	0.14	0.060	−0.31	0.13	0.017

Smooth Terms	edf		*p*	edf		*p*	edf		*p*
Age by sex (F)	3.58		< 0.001	4.43		< 0.001	3.61		< 0.001
Age by sex (M)	4.76		< 0.001	5.15		< 0.001	4.81		< 0.001
Adjusted *R* ^2^	0.636			0.596			0.59		

Response variable	Upper arm circumference			Chest circumference			Tail length		
Parametric coefficients	Estimate	SE	*p*	Estimate	SE	*p*	Estimate	SE	*p*
Intercept	8.76	0.07	< 0.001	23.87	0.19	< 0.001	49.85	0.40	< 0.001
Sex (M)	0.41	0.10	< 0.001	0.16	0.26	0.541	−0.79	0.54	0.147

Smooth terms	edf		*p*	edf		*p*	edf		*p*
Age by sex (F)	4.08		< 0.001	3.82		< 0.001	3.62		< 0.001
Age by sex (M)	3.93		< 0.001	5.06		< 0.001	3.89		< 0.001
Adjusted *R* ^2^	0.807			0.731			0.281		

Response variable	Maxillary canine length			Intermembral index		
Parametric coefficients	Estimate	SE	*p*	Estimate	SE	*p*
Intercept	7.09	0.13	< 0.001	65.46	0.39	< 0.001
Sex (M)	0.11	0.17	0.519	1.47	0.54	0.007

Smooth Terms	edf		*p*	edf		*p*
Age by sex (F)	6.23		< 0.001	1		0.0012
Age by sex (M)	6.65		< 0.001	1		< 0.001
Adjusted *R* ^2^	0.821			0.184		

*Note:* The parametric coefficient “Sex” refers to a difference in growth across development between the sexes. The smooth terms “Age by Sex” refer to the shape of each growth curve. A significant smooth term means that the line of the growth curve, or smooth, is significantly different from zero, but does not mean that there is a significant difference between the shape of the male and female curves.

Abbreviations: F, females; M, males.

**FIGURE 4:**
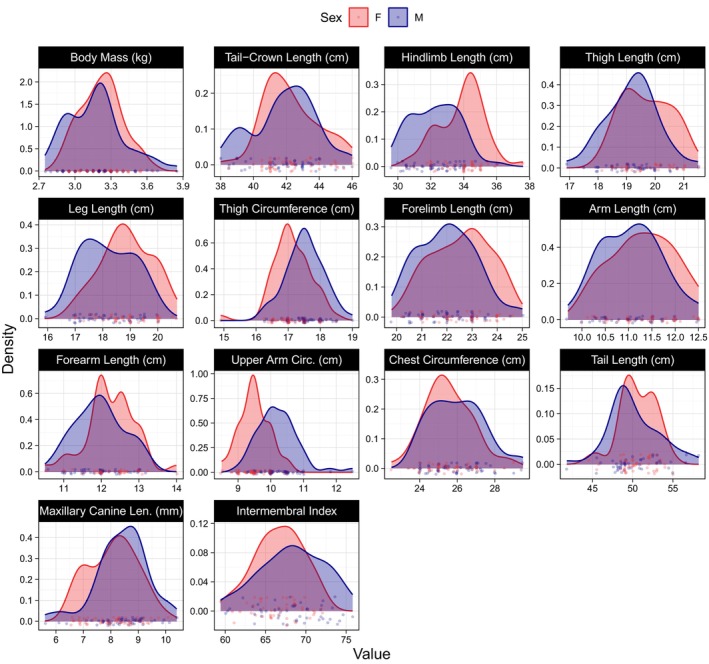
Sex‐specific density distributions of morphological trait values in adult Verreaux's sifaka. Only adults aged 6 years and older are included. Each point represents an individual measurement. All values were normally distributed within each sex.

While not significant in *t*‐tests, the fixed effect of sex was significant in the GAMMs for forelimb and forearm length and trended towards significance in arm length, with adult females exhibiting longer lengths in all three dimensions (Table 3). A significant fixed effect of sex in a GAMM with no significant difference in the *t*‐test indicated that male and female growth curves were significantly different in these traits, but that these traits were not sexually dimorphic in adulthood. Adult males and females exhibited no significant difference in body mass, tail‐crown length, chest circumference, tail length, or maxillary canine length in either *t*‐tests or GAMMs (Tables 2 and 3). Values in all traits were normally distributed for individuals aged 6 years and older (Figure 4).

## 4 Discussion

By examining sex differences beyond body mass and adulthood, we found that adult Verreaux's sifaka exhibited sexual size monomorphism in body mass but sexual dimorphism in adult size, growth duration, and growth rate across multiple other traits. Morphometrically, Verreaux's sifaka at Ankoatsifaka reach adulthood at age 6 years. Contrary to the fecundity advantage hypothesis and the reproductive constraints hypothesis, female sifaka did not exhibit an enlargement or reduction of overall shape compared to males. However, consistent with these hypotheses, female skeletal growth was completed by the onset of reproduction. Contrary to the male contest hypothesis, sifaka did not exhibit male‐biased dimorphism in most of the traits associated with fighting, nor did males exhibit longer or faster growth than females in most of these traits. Nevertheless, our results also do not support the idea that Verreaux's sifaka are simply monomorphic at adulthood or across development. We argue that our observed patterns of dimorphism are indeed consistent with the hypotheses that female reproduction and male contest competition influence adult size and growth trajectories, just in a more limited and previously unexpected pattern.

### 4.1 Female Reproduction

We did not find support for the fecundity advantage hypothesis (Darwin 1871; Gordon 2006b; Kuzawa and Bragg 2012; Püschel et al. 2025; Ralls 1976). Inconsistent with our predictions for this hypothesis, adult sifaka inhabiting Kirindy Mitea National Park exhibited sexual monomorphism in body mass. Females also did not exhibit a larger adult body size across 12 of our 14 morphological measurements (Table 2). These results suggest that female sifaka do not derive a reproductive benefit from having a larger overall body size relative to males. Consistent with our predictions, however, females completed growth by age 6 years, the average age of first reproduction (Bronikowski et al. 2016; Voyt et al. 2019; c.f., King et al. 2011). While some subadult female sifaka (ages 3–4 years) do give birth, their offspring do not survive (Richard et al. 2002; Voyt et al. 2019), suggesting that females derive reproductive benefits from their larger skeletal size and mass beginning ages 5 and 6 years, respectively. Further research is needed to determine whether these larger and older females produce bigger and more robust infants. Originally proposed for oviparous species (Darwin 1871; Shine 1988), the fecundity advantage hypothesis was extended to mammals by linking large female body size to the production of larger, more robust infants (Gordon 2006b; Kuzawa and Bragg 2012; Ralls 1976). However, large‐scale analyses using multiple species suggest that small body size is more advantageous for female primates, potentially due to increased energetic efficiency (Cassini 2020; Lindenfors 2002). More in‐depth, targeted analyses do support the fecundity advantage hypothesis for some primate species (e.g., *Saguinus mystax* : Gordon 2006a; hominins: Püschel et al. 2025), particularly in resource‐abundant environments where females can support larger body mass (Plavcan 2011; Ralls 1976).

We did not find support for the reproductive constraints hypothesis (Cassini 2020; Clutton‐Brock et al. 1977; Gordon 2006a; Lindenfors 2002). Female sifaka were smaller than males in only two of our 14 measurements, suggesting that overall female size is not reduced compared to male size to increase energetic efficiency in females. The forest in Kirindy Mitea National Park exhibits highly seasonal rainfall (Lewis and Axel 2019), photosynthetic activity (Axel et al. 2024), and sifaka food availability (Veilleux et al. 2024), in addition to droughts (Axel et al. 2024; Veilleux et al. 2024) and cyclones (Lewis and Bannar‐Martin 2012; Lewis and Rakotondranaivo 2011). This regular exposure to scarcity is expected to favor smaller size in females (Gordon 2006a; Plavcan 2011) and reduced female size has been reported in populations that experience fluctuations in resource availability (e.g., *Cercopithecus aethiops* : Gordon 2006a; *Erythrocebus patas* : Rowell 1977). While our finding that female sifaka completed growth by reproductive onset is consistent with the reproductive constraints hypothesis, the observation that the infants of younger, and thus smaller, females do not survive is not (Richard et al. 2002; Voyt et al. 2019). Whether this lower fecundity is a result of a smaller body size, age, inexperience, prioritizing growth over reproduction in energetic tradeoffs, or another variable remains to be tested, however.

We did find female‐biased sexual dimorphism in traits consistent with the hypothesis that female reproduction drives some sex differences in Verreaux's sifaka. Adult females exhibited significantly longer hindlimbs and thighs than males (Table 2 and Figure 1C). This greater female hindlimb length is responsible for females exhibiting a lower intermembral index than males across development and in adulthood (Figure 1N). Sifaka are thigh‐powered leapers (Gebo and Dagosto 1988): their long hindlimbs, and in particular their long thighs, allow them to achieve greater acceleration, force, takeoff velocity, and distance in leaps that can exceed 10 m (Anemone 1990; Demes et al. 1996, 1998; Jolly 1966; Napier and Walker 1967). Greater hindlimb length thus allows females to leap longer distances than males, and perhaps, compensate for the locomotor consequences of dorsal infant carrying. Importantly, sex differences in hindlimbs did not arise until after age 4 years (Figures 1C and 3C), when a female might first give birth. The timing of this divergence suggests that female‐biased dimorphism in hindlimb and thigh length is related to reproductive activities, such as infant carrying. However, this growth pattern could also have non‐adaptive explanations. Estrogen levels, which vary between males and females and increase at reproductive maturity, strongly influence long bone growth, pelvis growth, and epiphyseal closure (Cutler 1997; Dunsworth 2020; Singh et al. 2010). That sex differences in hindlimb length arise at reproductive maturity may thus reflect hormonal sex differences at puberty instead of direct selection for hindlimb length (Dunsworth 2020), however, no hormonal data were available to test this possibility.

### 4.2 Contest Competition

We did not find strong support for the male contest hypothesis. Adult males did not exhibit longer limbs, longer maxillary canines, or larger body mass than females, nor did they grow at faster rates or longer durations in these traits (Table 2 and Figure 3). Indeed, contrary to previous research showing that male hindlimb length is under positive selection in Verreaux's sifaka (Lawler et al. 2005), we found that adult males had significantly shorter hindlimbs and thighs than females (Table 2). According to the male contest competition hypothesis, sexual monomorphism is expected in species where levels of male–male and female–female intrasexual contest competition are equal (Andersson and Iwasa 1996; Emlen and Wrege 2004; Rosvall 2011). In primates, monogamous species are thus expected to exhibit the highest rates of sexual size monomorphism (Cassini 2020; Clutton‐Brock et al. 1977; Harvey et al. 1978; Plavcan and van Schaik 1997), but Verreaux's sifaka mate polygynandrously (Brockman 1999). Nonetheless, based on our predictions, our results suggest that male sifaka do not experience greater levels of intrasexual contest competition than female sifaka.

In support of the male contest hypothesis, however, adult male sifaka had significantly larger upper arm and thigh circumferences than adult females (Table 2). As thigh‐powered leapers (Gebo and Dagosto 1988), increased thigh muscle mass improves male leaping ability, though the extent of the effect of increased thigh mass in males relative to increased thigh and hindlimb length in females is unclear. Lengthy chases during male–male competition may select positively for thigh muscle mass in males, especially with male body mass under balancing selection (Lawler et al. 2005), and greater upper arm and thigh muscle mass likely aid males in the wrestling stage of fights. Indeed, thigh circumference correlates with paternity in Verreaux's sifaka (Lawler et al. 2005). In further support of the male contest hypothesis, male sifaka exhibited longer growth duration in both thigh and upper arm circumferences compared to females, attaining adult musculature size by ages 5–6 years, respectively (Table 4 and Figure 1). Age 5–6 years is the age at which males can first become dominant among male group members, attempt an aggressive group takeover, and first be treated as competitors in sperm competition (Brockman et al. 2001; Bueno and Lewis 2024; Leimberger and Lewis 2017; Richard et al. 1993). The causal direction of the growth pattern in musculature is unclear, however, and these behavioral patterns may alternatively limit male growth. Extended male growth in traits related to fighting is common in sexually dimorphic primates, wherein males delay full development of competition‐related traits until they start to fully engage in contest competition for mates (e.g., Leigh 1992, 1995; Setchell and Dixson 2002; Setchell et al. 2001; Turcotte et al. 2022).

**TABLE 4:** Age (in years) at adult size in Verreaux's sifaka for 14 measurements.

Measurement	Female	Male
Body mass	6.2	6.3
Tail‐crown length	5.1	4.0
Hindlimb length	4.3	3.2
Thigh length	4.1	3.1
Leg length	2.6	2.8
Thigh circumference	4.5	5.2
Forelimb length	4.2	3.2
Arm length	3.1	3.2
Forearm length	3.6	3.3
Upper arm circumference	4.3	6.3
Chest circumference	5.2	3.4^a^
Tail length	3.2	3.2
Maxillary canine length	3.6	3.2

^a^ Based on visual examination of growth curves (Figure 1K), males and females appear to follow the same trajectory in chest growth.

While body mass is the most commonly studied contest‐related physical trait in male primates (e.g., Cassini 2020; Clutton‐Brock and Harvey 1977; Kappeler 1990; Leigh 1992; Leutenegger and Kelly 1977; Plavcan 2012), sexual selection also targets other traits associated with fighting performance in primates and other mammals, including muscle mass (e.g., *C. familiaris* : Pasi and Carrier 2003; *G. gorilla* : Barel Hooge et al. 2024; Zihlman and McFarland 2000; *M. musculus* : Cooper et al. 2020; *O. crassicaudatus* : Leigh et al. 2024), limb length (e.g., *G. tippelskirchi* : Cavener et al. 2024; *G. gorilla* : Breuer et al. 2007; macropodids: Richards et al. 2015), and canine size (e.g., anthropoids: Leutenegger and Kelly 1977; canids: Gittleman and Valkenburgh 1997; *H. amphibius* : Shannon et al. 2021; *M. sphinxus* : Leigh et al. 2008; Moschidae: Jarman 1983; platyrrhines: Kay et al. 1988), as well as more unique, species‐specific traits such as neck width in Masai giraffes ( *G. tippelskirchi* : Cavener et al. 2024) and melon size in bottlenose whales ( *Hyperoodon ampullatus* : Gowans and Rendell 1999). Even primates with male‐biased dimorphism in body mass employ multiple tactics during fights, including biting and chasing ( *Cebus capucinus* : Gros‐Louis et al. 2003; *Macaca mulatta* : Chancellor and Isbell 2008; *M. sinica* : Dittus and Ratnayeke 1989; *Pan paniscus* : Cordoni et al. 2023; *Semnopithecus schistaceus* : Feder et al. 2019). While body mass can be a decisive tool in male–male contest competition (Plavcan 2001, 2012), males across species fight using a suite of traits, and the weapons, tactics, and associated sexual dimorphism in this suite are likely to vary across species (Jarman 1983; Morris and Carrier 2016; Oxnard 1983).

Across anthropoids, levels of inter‐male competition correlate with levels of male‐biased sexual size dimorphism in body mass (Cassini 2020; Leutenegger and Cheverud 1982; Plavcan 2001; Weckerly 1998). Lemurs do not fit this pattern, with strong inter‐male competition present in sexually size‐monomorphic species (Kappeler 1990; Weckerly 1998). To try to resolve this purported paradox, researchers have hypothesized that sexual selection may act on male traits, such as agility, instead of body size (Clutton‐Brock 1985; Kappeler 1990; Lawler et al. 2005). While male sifaka's increased thigh muscle size is expected to improve their agility and leaping (Demes et al. 1998), their large upper arm muscle size probably does not, especially relative to leg size. The larger upper arm muscles in male sifaka are instead more likely a measure of male fighting ability (Li et al. in revision). Large thigh muscle size probably also contributes to male fighting ability, but its contribution to grappling versus leaping is difficult to disentangle. Sifaka thus do appear to fit the expected primate pattern of male competition correlating with sexual size dimorphism but with muscle size instead of body mass dimorphism. Analyzing sexual dimorphism as just one metric (e.g., body mass) can be useful, particularly within clades, but may limit interspecific comparisons because the traits that influence competitive ability may be different but analogous across species (e.g., Kay et al. 1988). We therefore recommend that researchers remain aware of these types of interspecific differences, and exercise caution when interpreting broad comparative patterns based on characteristics that may present as species‐specific analogs rather than direct homologs. Future comparative research could examine combined measures of fighting ability using methods such as geometric mean (e.g., Cobb and O'Higgins 2007; Wang et al. 2007), intermembral index, or PCA (e.g., Li et al. in revision).

### 4.3 The Meaning of Monomorphism

While Verreaux's sifaka exhibited sex differences in several adult traits and growth patterns, most adult traits showed no sexual dimorphism (Table 2), and males and females followed monomorphic growth trajectories in half of the traits examined (Figure 1 and Table 4). This pattern could be interpreted as evidence that male and female sifaka do not face different selective pressures in any of these traits. However, the absence of sexual dimorphism does not necessitate the absence of sex‐specific selection because monomorphic traits might arise when different selective pressures acting on males and females converge on similar adult phenotypes (Gordon 2006a, 2006b; Jarman 1983; Plavcan 2011). In sifaka, female body mass may be constrained to improve energetic efficiency during reproduction, as seen in other primates living in fluctuating environments (e.g., Gordon 2006a), whereas male body mass may be constrained by different pressures (e.g., selection for agility: Clutton‐Brock 1985; Kappeler 1990; Lawler et al. 2005), resulting in sexual monomorphism in body mass. Female‐biased bimaturism in two traits that are monomorphic at adulthood (tail‐crown and forelimb length; Table 4) suggests males and females may face different pressures on skeletal growth. While theoretically unexpected (e.g., Leigh 1992, 1995), similar patterns of sexually dimorphic trajectories in monomorphic adult traits have been reported in other primates (body mass of *Eulemur macaco, E. mongoz, Microcebus murinus*: Tennenhouse 2015; neurocranium of *M. mulatta* : Wang et al. 2007). These results exemplify the importance of incorporating developmental data when examining sexual dimorphism, because evidence of these sex differences vanish by adulthood.

Furthermore, sexual dimorphism in a trait does not necessarily imply that the smaller sex is under balancing or negative selection in that trait. Males and females can face different selective pressures on the same trait (Gordon 2006a, 2006b; Oxnard 1983; Plavcan 2001, 2011). These sex‐specific pressures can cause sexual dimorphism when they differ in type, mechanism, and level of intensity. In Verreaux's sifaka, genetic analyses indicate positive selection on male thigh length (Lawler et al. 2005), however, in our analyses, adult females exhibited longer thighs and hindlimbs than males (Table 2). Combined, these results suggest that male and female Verreaux's sifaka experience different positive selective pressures on thigh length. A similar pattern is evident in hominins, where both sexes experience positive selection on body mass despite male‐biased dimorphism in mass (Püschel et al. 2025). Together, these findings caution against assuming that sexual monomorphism reflects identical selective pressures, or that sexual dimorphism necessarily arises from opposing selection on male and female size (Gordon 2006a, 2006b; Oxnard 1983; Plavcan 2001, 2011).

## 5 Conclusion

Sexual size monomorphism in adult body mass does not necessitate monomorphism across all non‐reproductive traits and growth trajectories in primates (e.g., Lawler et al. 2005; Leigh 1992; Tennenhouse 2015). Mating competition and reproductive energetics can impose selective pressures on morphological traits that are particular to the ecological and social environment of each species (e.g., Leigh 1995; Leigh and Shea 1996; Plavcan 2001, 2012; Plavcan and van Schaik 1997). Consequently, researchers should be circumspect when interpreting interspecific comparisons of traits because the lack of dimorphism in one trait in a species does not equal a lack of effect of that selective pressure on the morphology of that species. Furthermore, sex differences in growth trajectories and non‐mass morphometric traits are likely present across other primates, including species that are both sexually monomorphic and dimorphic in adult body mass (e.g., Leigh and Shea 1995; Tennenhouse 2015), but published data are limited. Noninvasive methods (e.g., laser photogrammetry) have expanded the ability of researchers to collect morphometric and growth data in wild populations to answer similar questions in more species (e.g., *G. gorilla* : Breuer et al. 2007; *Macaca assamensis* : Anzà et al. 2022; *Tursiops truncatus* : Cheney et al. 2018). A greater comparative dataset, for instance, would clarify whether traits, such as pelvis shape (Leutenegger 1974; Moffett 2017) and leg length (this study), are associated with particular forms of locomotion (e.g., bipedality, vertical clinging and leaping) or common female adaptations to reproduction. Examining dimorphism beyond body mass and beyond adulthood across species provides the opportunity to delve more deeply into questions about sex differences and ontogeny.

## Author Contributions

**Gabrielle L. Bueno:** conceptualization, investigation, writing – original draft, methodology, visualization, writing – review and editing, software, formal analysis, project administration, validation, data curation. **Stacey R. Tecot:** funding acquisition, writing – review and editing, supervision. **Rebecca J. Lewis:** conceptualization, investigation, funding acquisition, writing – review and editing, project administration, data curation, supervision, resources.

## Funding

This work was supported by Primate Conservation Inc., Donald D. Harrington Fellows Program, University of Texas at Austin, Leakey Foundation, National Science Foundation (BES 1719654, DGE 2137420).

## Conflicts of Interest

The authors declare no conflicts of interest.

## Supporting information


**Table S1:** Description of all sifaka included in analyses by subject identity, sex, age at capture(s), total number of times an individual was captured between 2006 and 2019, and whether year of birth was known or assigned. Please see Methods for explanation of when and how year of birth was assigned. Totals for each category are included at the end, with totals separated by total number of individuals and total number of times these individuals were captured. “Total # of Individuals” describes the number of individual sifaka included in each age category. “Total # of Captures” describes the number of datapoints across all individuals in each age category, because some individuals were captured and measured multiple times throughout the study period (maximum number of recaptures = 8). Please note that for *t*‐tests examining levels of sexual dimorphism in adults 6 years and older, morphometric data was averaged across captures within each individual. F: Females, M: Males.
**Table S2:** Demographic makeup of adult sifaka samples included in analyses, separated by whether year of birth was known or assigned. Please see Methods for additional details on how and when year of birth was assigned. Because some individuals were captured and measured multiple times throughout the study period (maximum number of recaptures = 8), we report both the total number of individual sifaka and the total number of captures. Please note that for *t*‐tests examining levels of sexual dimorphism in adults 6 years and older, morphometric data was averaged within each individual.

## Data Availability

The data that support the findings of this study are available from the corresponding author upon reasonable request.
