# Supplementary material for: Beyond Body Mass, Beyond Adulthood: The Ontogeny of Sexual Size Monomorphism
Source: Am J Biol Anthropol. 2026 May 4;190:e70257. doi: 10.1002/ajpa.70257 (PMC13139752; doi:10.1002/ajpa.70257)
Supplement: Supplementary file 1 — Table S1: Description of all sifaka included in analyses by subject identity, sex, age at capture(s), total number of times an individual was captured between 2006 and 2019, and whether year of birth was known or assigned. Please see Methods for explanation of when and how year of birth was assigned. Totals for each category are included at the end, with totals separated by total number of individuals and total number of times these individuals were captured. “Total # of Individuals” describes the number of individual sifaka included in each age category. “Total # of Captures” describes the number of datapoints across all individuals in each age category, because some individuals were captured and measured multiple times throughout the study period (maximum number of recaptures = 8). Please note that for t‐tests examining levels of sexual dimorphism in adults 6 years and older, morphometric data was averaged across captures within each individual. F: Females, M: Males. Table S2: Demographic makeup of adult sifaka samples included in analyses, separated by whether year of birth was known or assigned. Please see Methods for additional details on how and when year of birth was assigned. Because some individuals were captured and measured multiple times throughout the study period (maximum number of recaptures = 8), we report both the total number of individual sifaka and the total number of captures. Please note that for t‐tests examining levels of sexual dimorphism in adults 6 years and older, morphometric data was averaged within each individual. [file AJPA-190-e70257-s001.docx]

**Supplemental Material**

**Permit Information**

All data were collected under with permits from both Madagascar National Parks and the University of Texas at Austin IACUC.

Madagascar National Parks permit numbers: 84/06, 159/06, 049/08, 30/09, 38/09, 175/10, 131/11, 002/12, 235/12, 076/13, 003/14, 203/14, 100/15, 448/DBA/15/FR, 17/16, 133/17, 55/18, 310/18, 226/19.

IACUC Permits: 05101801, 08110301, AUP-2011-00143, AUP-2014-00361, AUP-2017-00152, AUP-2020-00143.

| **Supplementary Table 1.** Description of all sifaka included in analyses by subject identity, sex, age at capture(s), total number of times an individual was captured between 2006 and 2019, and whether year of birth was known or assigned. Please see Methods for explanation of when and how year of birth was assigned. Totals for each category are included at the end, with totals separated by total number of individuals and total number of times these individuals were captured. “Total # of Individuals” describes the number of individual sifaka included in each age category. “Total # of Captures” describes the number of datapoints across all individuals in each age category, because some individuals were captured and measured multiple times throughout the study period (maximum number of recaptures = 8). Please note that for t-tests examining levels of sexual dimorphism in adults 6 years and older, morphometric data was averaged across captures within each individual. F: Females, M: Males. | | | | | | | | |
| --- | --- | --- | --- | --- | --- | --- | --- | --- |
| **Subject ID** | **Age 1** | **Age 2** | **Age 3** | **Age 4** | **Age 5** | **Age 6+** | **Total # of Captures** | **Year of Birth Status** |
| F1 |  |  |  | 1 |  |  | 1 | Known |
| F2 |  |  |  |  |  | 1 | 1 | Estimated |
| F3 |  |  |  |  |  | 6 | 6 | Estimated |
| F4 | 1 |  |  |  |  |  | 1 | Known |
| F5 |  |  |  |  |  | 6 | 6 | Estimated |
| F6 |  |  |  |  |  | 7 | 7 | Estimated |
| F7 |  |  |  |  | 1 | 3 | 4 | Known |
| F8 |  |  |  |  |  | 8 | 8 | Estimated |
| F9 | 1 |  |  |  |  |  | 1 | Known |
| F10 |  |  |  | 1 |  |  | 1 | Known |
| F11 |  |  |  |  | 1 | 2 | 3 | Known |
| F12 |  |  |  |  |  | 3 | 3 | Estimated |
| F13 |  |  |  |  |  | 3 | 3 | Estimated |
| F14 | 1 |  | 1 |  |  |  | 2 | Known |
| F15 | 1 |  |  |  |  |  | 1 | Known |
| F16 |  |  |  |  |  | 2 | 2 | Known |
| F17 | 1 |  |  |  |  |  | 1 | Known |
| F18 | 1 |  | 1 |  |  |  | 2 | Known |
| F19 | 1 |  |  |  |  |  | 1 | Known |
| F20 |  |  |  |  |  | 3 | 3 | Estimated |
| F21 | 1 |  | 1 |  | 1 | 1 | 4 | Known |
| F22 | 1 |  |  |  |  |  | 1 | Known |
| F23 | 1 |  |  |  |  |  | 1 | Known |
| F24 | 1 |  |  |  |  |  | 1 | Known |
| F25 | 1 |  |  |  |  |  | 1 | Known |
| F26 |  |  |  |  | 1 |  | 1 | Known |
| F27 | 1 | 1 | 1 |  |  |  | 3 | Known |
| F28 |  | 1 |  | 1 |  |  | 2 | Known |
| F29 |  |  |  |  |  | 5 | 5 | Estimated |
| F30 | 1 |  |  | 1 |  | 2 | 4 | Known |
| F31 | 1 |  |  |  |  |  | 1 | Known |
| F32 | 1 |  |  | 1 |  |  | 2 | Known |
| F33 | 1 |  |  |  |  |  | 1 | Known |
| F34 |  | 1 |  |  |  | 5 | 6 | Known |
| F35 | 1 |  | 1 |  |  |  | 2 | Known |
| F36 |  |  | 1 |  |  |  | 1 | Known |
| F37 | 1 | 1 |  |  |  | 3 | 5 | Known |
| F38 | 1 |  |  |  |  |  | 1 | Known |
| F39 |  |  |  |  |  | 2 | 2 | Estimated |
| M1 | 1 | 1 |  |  |  |  | 2 | Known |
| M2 | 1 |  |  |  |  |  | 1 | Known |
| M3 |  |  |  | 1 |  | 2 | 3 | Known |
| M4 |  |  | 1 |  |  | 1 | 2 | Known |
| M5 | 1 |  | 1 |  |  | 1 | 3 | Known |
| M6 | 1 |  |  |  | 1 |  | 2 | Known |
| M7 |  |  |  |  |  | 1 | 1 | Estimated |
| M8 |  |  | 1 |  |  |  | 1 | Known |
| M9 |  |  |  |  |  | 4 | 4 | Estimated |
| M10 |  |  | 1 | 1 |  | 2 | 4 | Known |
| M11 |  |  | 1 |  |  |  | 1 | Known |
| M12 | 1 |  | 1 |  |  |  | 2 | Known |
| M13 | 1 |  |  |  |  |  | 1 | Known |
| M14 |  |  |  |  |  | 1 | 1 | Estimated |
| M15 | 1 |  | 1 |  |  |  | 2 | Known |
| M16 |  | 1 |  |  |  |  | 1 | Known |
| M17 | 1 | 1 |  | 1 |  | 1 | 4 | Known |
| M18 |  |  |  |  |  | 2 | 2 | Estimated |
| M19 |  | 1 |  | 1 |  | 1 | 3 | Known |
| M20 |  | 1 |  | 1 |  | 4 | 6 | Known |
| M21 | 1 |  |  |  |  |  | 1 | Known |
| M22 | 1 | 1 |  | 1 | 1 | 2 | 6 | Known |
| M23 |  |  |  |  |  | 4 | 4 | Estimated |
| M24 |  |  |  |  |  | 4 | 4 | Estimated |
| M25 | 1 | 1 |  | 1 |  |  | 3 | Known |
| M26 |  |  |  |  |  | 4 | 4 | Estimated |
| M27 |  |  |  |  |  | 1 | 1 | Estimated |
| M28 |  |  | 1 |  |  |  | 1 | Known |
| M29 | 1 |  |  |  |  | 1 | 2 | Known |
| M30 |  |  |  |  |  | 1 | 1 | Estimated |
| M31 | 1 |  |  |  |  |  | 1 | Known |
| M32 |  |  |  |  |  | 1 | 1 | Estimated |
| M33 | 1 |  |  |  |  |  | 1 | Known |
| M34 |  |  |  |  |  | 2 | 2 | Estimated |
| M35 |  |  |  |  |  | 4 | 4 | Estimated |
| M36 |  |  |  |  |  | 1 | 1 | Estimated |
| M37 |  |  |  |  |  | 3 | 3 | Estimated |
| M38 | 1 |  | 1 |  |  |  | 2 | Known |
| M39 |  | 1 |  | 1 | 1 |  | 3 | Known |
| M40 |  |  |  |  |  | 3 | 3 | Estimated |
| M41 | 1 |  |  |  |  |  | 1 | Known |
| M42 | 1 |  |  |  |  |  | 1 | Known |
| M43 | 1 | 1 |  |  |  |  | 2 | Known |
| M44 | 1 |  | 1 |  |  |  | 2 | Known |
| M45 | 1 | 1 |  |  |  |  | 2 | Known |
| M46 |  | 1 |  |  |  |  | 1 | Known |
| M47 | 1 |  |  |  |  |  | 1 | Known |
| M48 | 1 |  |  | 1 |  |  | 2 | Known |
| M49 |  |  |  |  |  | 3 | 3 | Estimated |
| M50 | 1 |  |  |  |  |  | 1 | Known |
| **Total # of Captures** | 43  N♀=20 | 15  N♀=4 | 16  N♀=6 | 14  N♀=5 | 7  N♀=4 | 116  N♀=62 | 211  N♀=101 |  |
| **Total # of Individuals** | 43  N♀=20 | 15  N♀=4 | 16  N♀=6 | 14  N♀=5 | 7  N♀=4 | 42  N♀=17 | 89  N♀=39 |  |

| **Supplementary Table 2.** Demographic makeup of adult sifaka samples included in analyses, separated by whether year of birth was known or assigned. Please see Methods for additional details on how and when year of birth was assigned. Because some individuals were captured and measured multiple times throughout the study period (maximum number of recaptures = 8), we report both the total number of individual sifaka and the total number of captures. Please note that for t-tests examining levels of sexual dimorphism in adults 6 years and older, morphometric data was averaged within each individual. | | | | | |
| --- | --- | --- | --- | --- | --- |
|  | **Known Birth Year** | | **Assigned Birth Year** | | **TOTAL** |
|  | *Females* | *Males* | *Females* | *Males* |  |
| **Number of individual adults** | 7 | 9 | 10 | 16 | 42 |
| **Total times captured (including recaptures)** | 18 | 15 | 44 | 39 | 116 |
